# Complete chloroplast genome sequence of *Solanum mochiquense*, one of the tuber-bearing potato relatives

**DOI:** 10.1080/23802359.2024.2432357

**Published:** 2024-11-21

**Authors:** Tae-Ho Park

**Affiliations:** Department of Horticulture, Daegu University, Gyeongsan, South Korea

**Keywords:** cpDNA, Peruvian species, plastom genome, phylogenetic tree

## Abstract

*Solanum mochiquense* is one of the wild tuber-bearing *Solanum* species belonging to the Solanaceae family. In this study, the chloroplast genome sequence of the species was completed with Illumina sequencing technology. The total length of the chloroplast genome is 155,547 bp with a GC content of 37.87%. It comprises a large single copy (LSC) region of 85,941 bp, a small single copy (SSC) region of 18,382 bp, and two inverted repeat regions (IRa and IRb) of 25,612 bp. Additionally, 158 functional genes in the genome were identified, including 105 protein-coding genes, eight ribosomal RNA genes, and 45 transfer RNA genes. Phylogenetic analysis revealed that *S. mochiquense* is grouped into a large clade with other *Solanum* species including cultivated potatoes (*S. tuberosum*). This study provides useful genomic information for future breeding and evolutionary studies of *S. mochiquense* and other *Solanum* species.

## Introduction

*Solanum mochiquense* Ochoa 1822 is one of the wild Peruvian tuber-bearing species of cultivated potatoes (*Solanum tuberosum*) (Correll 1962). Due to its resistance to diverse diseases caused by *Erwinia carotovora*, *Synchytrium endobioticum*, potato viruses, the nematode *Meloidogyne*, *Phytophthora infestans*, etc. (Ochoa 1998; Ruíz de Galarreta et al. 1998; Smilde et al. 2005), it could be one of the great resources for potato breeding. However, it is a diploid, and its endosperm balance number (EBN) of 1 is different from those (tetraploid and EBN 2) of *S. tuberosum*, which theoretically does not allow direct cross between the two different species for potato breeding (Hawkes 1990; Ortiz and Ehlenfeldt 1992; Cho et al. 1997). That inconvenience caused by the different ploidy levels and EBNs must be overcome with one of several methods, such as bridged crosses, polyploidization or diploidization, somatic hybridization, or genetic modification (Cho and Park 2014). To introduce novel traits from *S. mochiquense* into *S. tuberosum*, intraspecific hybrids and transgenic lines were developed and analyzed for late blight resistance (Smilde et al. 2005; Jones et al. 2010; Aguilera-Galvez et al. 2018), but somatic hybridization has not been used with *S. mochiquense* for potato breeding. In our contemporary researches, therefore, somatic hybridization has been performed between *S. mochiquense* and *S. tuberosum* (data not shown). After obtaining somatic hybrids, it is important to identify the nuclear and cytoplasmic genome composition, because somatic hybrids create novel variability both in the nuclear DNA and cytoplasmic DNA of the mitochondria and chloroplast during somatic fusion between two sexually incompatible species (Guo et al. 2004; Iovene et al. 2007; Tiwari et al. 2014; Cho et al. 2016; Cho and Park 2016). Cytogenetic analysis, molecular markers, and certain genes have been used to identify the nuclear genome compositions (Williams et al. 1990; Yamada et al. 1998; Spooner et al. 2008; Pendinen et al. 2012; Ono et al. 2016), but the contribution of a plastid genome with *Solanum* species during somatic hybridization has rarely been studied. Therefore, this study completed the chloroplast genome sequence of *S. mochiquense* to develop molecular markers which will be used to identify chloroplast genome composition after obtaining hybrids *via* somatic hybridization.

## Materials and methods

**Figure 1.**
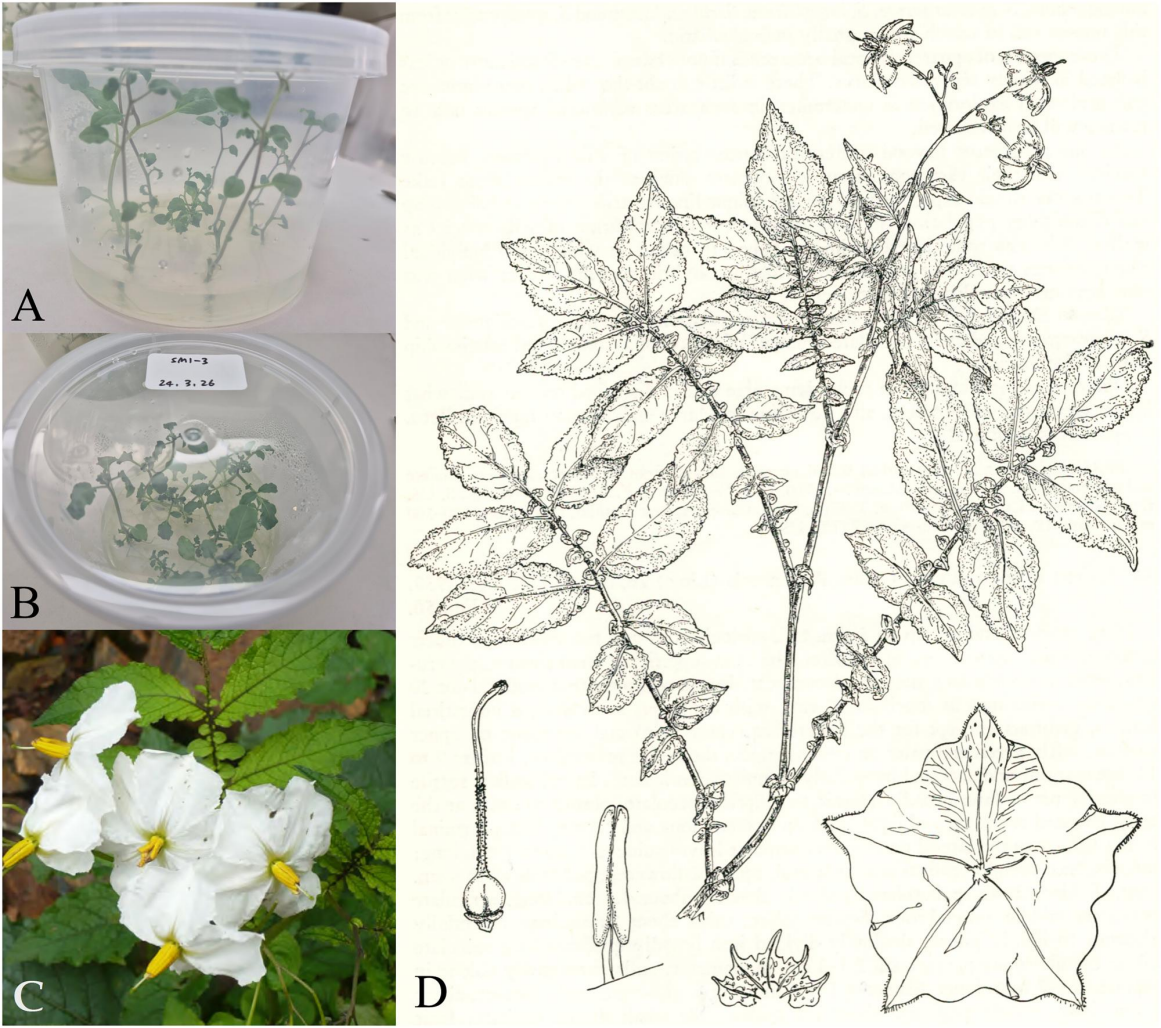
The species reference images for *S. mochiquense*. A and B: *In vitro* plants of *S. mochiquense* (SM1-3) used to complete the chloroplast genome sequence of *S. mochiquense* for this study. C: Wild *S. mochiquense* plant (Rodríguez et al. 2020). D: Illustration of *S. mochiquense* (Correll 1962). The characteristics of the species are followed; the stems are erect, ascending, tall, and stoloniferous with a slender and puberulent surface. Tubers are globose and white and their eyes are superficial. The leaves are long and glabrous with numerous several-sized interstitial leaflets.

*S. mochiquense* plants (PI338616) were provided by Highland Agriculture Research Institute, Republic of Korea (37°68′05.4"N 128°73′09.1"E) (Figure 1), and a specimen was deposited in the National Agrobiodiversity Center, Republic of Korea (http://genebank.rda.go.kr/, Ji-Hong Cho, jhcho0108@korea.kr) under voucher number IT301493.

Total genomic DNA was extracted with *in vitro* plants of *S. mochiquense* according to the manufacturer’s instructions (RBC Bioscience, New Taipei City, Taiwan). Basically, the Phyzen bioinformatics pipeline (Kim et al. 2015) was used for chloroplast genome sequencing of the species. The library was constructed based on the Paired-end (PE) standard protocol (Illumina, San Diego, USA) and PE sequencing was performed on the Illumina HiSeq2000 platform. Clean reads were assembled using the dnaLCW method *via* the CLC *de novo* assembly program in CLC assembly cell package version 4.2.1 (CLC Inc, Aarhus, Denmark). The structure and sequence of the assembled chloroplast genome were analyzed along with results from a BLASTN search of the National Center for Biotechnology Information (NCBI) database and BLASTZ analysis (Schwartz et al. 2003) using the *S. sogarandinum* complete chloroplast genome (GenBank accession number MH021551) as a reference. Chloroplast genome annotation was performed by using the GeSeq program (Tillich et al. 2017). The circular genome map and the schematic map of the trans- and cis-splicing genes were generated using the CPGView software (Liu et al. 2023, http://www.1kmpg.cn/cpgview/).

Phylogenetic analysis was conducted using the 71 chloroplast coding sequences of *S. mochiquense* and 43 published species belonging to the Solanaceae family obtained from NCBI by using a maximum likelihood method with a general time reversible model and 1,000 bootstrap options in MEGA11 (Tamura et al. 2021).

## Results and discussion

**Figure 2.**
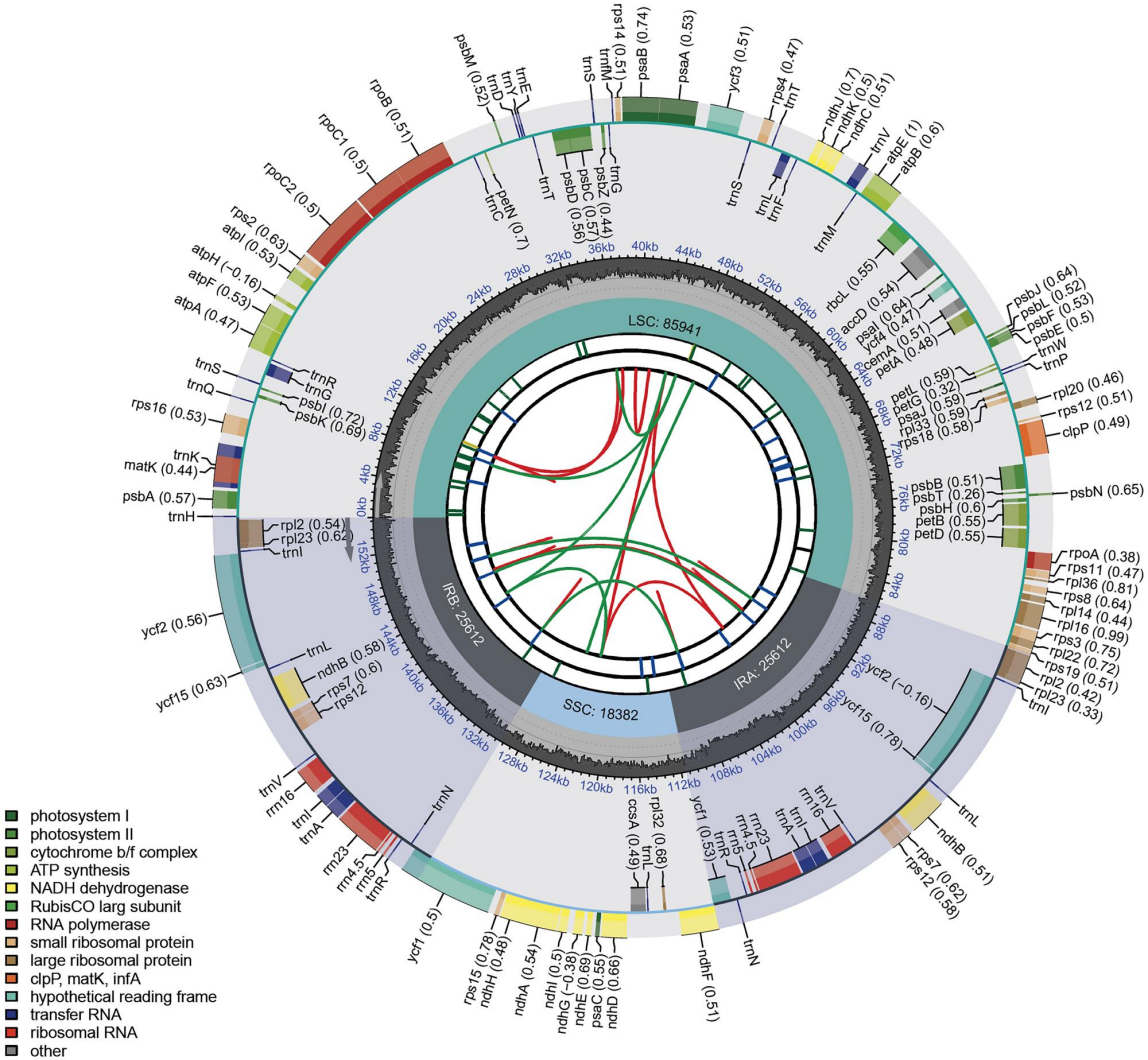
Schematic map of the overall features in the *S. mochiquense* chloroplast genome. The map contains six tracks. From the center outward, the first track shows the dispersed repeats, which consist of direct and palindromic repeats, connected with red and green arcs. The second track shows long tandem repeats as short blue bars. The third track shows short tandem repeats or microsatellite sequences as short bars with different colors. The colors, the type of repeat they represent, and the description of the repeat types are as follows. Black: complex repeat; green: repeat unit size = 1; yellow: repeat unit size = 2. The small single-copy (SSC), inverted repeat (IRa and IRb), and large single-copy (LSC) regions are shown on the fourth track. The GC content along the genome is plotted on the fifth track. The genes are shown on the sixth track. The optional codon usage bias is displayed in parentheses after the gene name. Genes are color-coded by functional classification. The transcription directions for the inner and outer genes are clockwise and anticlockwise, respectively. The functional classification of the genes is shown in the bottom left corner.

In total, approximately 2.65 Gbp of raw data were obtained and approximately 2.00 Gbp of clean reads were assembled. The average coverage was 843.74, and mapped read depth through the whole region was more than 400 (Supplementary Figure 1). The whole chloroplast genome sequence of *S. mochiquense* is 155,547 bp in length with a typical double-stranded loop structure. As chloroplast genome sequences of diverse *Solanum* species including cultivated potatoes (*S. tuberosum*) and wild *Solanum* species were identified (listed in Jo and Park 2024), the total length, structure, and GC content of the *S. mochiquense* chloroplast genome are highly similar with those of other *Solanum* species. Its structure is typically quadripartite and is divided into four regions consisting of a large single copy (LSC) region of 85,941 bp, a small single copy (SSC) region of 18,382 bp, and two inverted repeat regions (IRa and IRb) of 25,612 bp. The overall GC content was 37.87%. The BLASTN search results showed that the *S. mochiquense* sequence has a strong similarity (99.8% or more) to those of *S. sogarandinum* (99.86%), *S. blanco-galdosii* (99.86%), *S. cajamarquense* (99.82%), *S. multiinterruptum* (99.80%), and *S. augustii* (99.80%) which are also diploid (2n = 2x = 24) and originate from Peru (Ochoa 2004). The *S. mochiquense* chloroplast genome contains a total of 158 genes with an average size of 583.4 bp, including 105 protein-coding genes, 45 transfer RNA genes, and eight ribosomal RNA genes, with average sizes of 765.0 bp, 61.8 bp, and 1,133.3 bp, respectively (Figure 2). Eleven protein coding genes, nine tRNA genes, and four rRNA genes are duplicated in the IR regions. The *rps12* gene is a trans-splicing gene (Supplementary Figure 2A) and 13 genes (*rps16*, *atpF*, *rpoC1*, *ycf3*, *clpP*, *petB*, *petD*, *rpl16*, *rpl2*, *ndhB*, *ndhA*, *ndhB*, and *rpl2*) are cis-splicing genes (Supplementary Figure 2B). These results are exactly same as those of *S. iopetalum* (Park 2023a) and gene features are typically identical to those of higher plants. The results of chloroplast genome assembly and annotation were submitted to GenBank (http://www.ncbi.nlm.nih.gov/) under accession number MZ233589.

**Figure 3.**
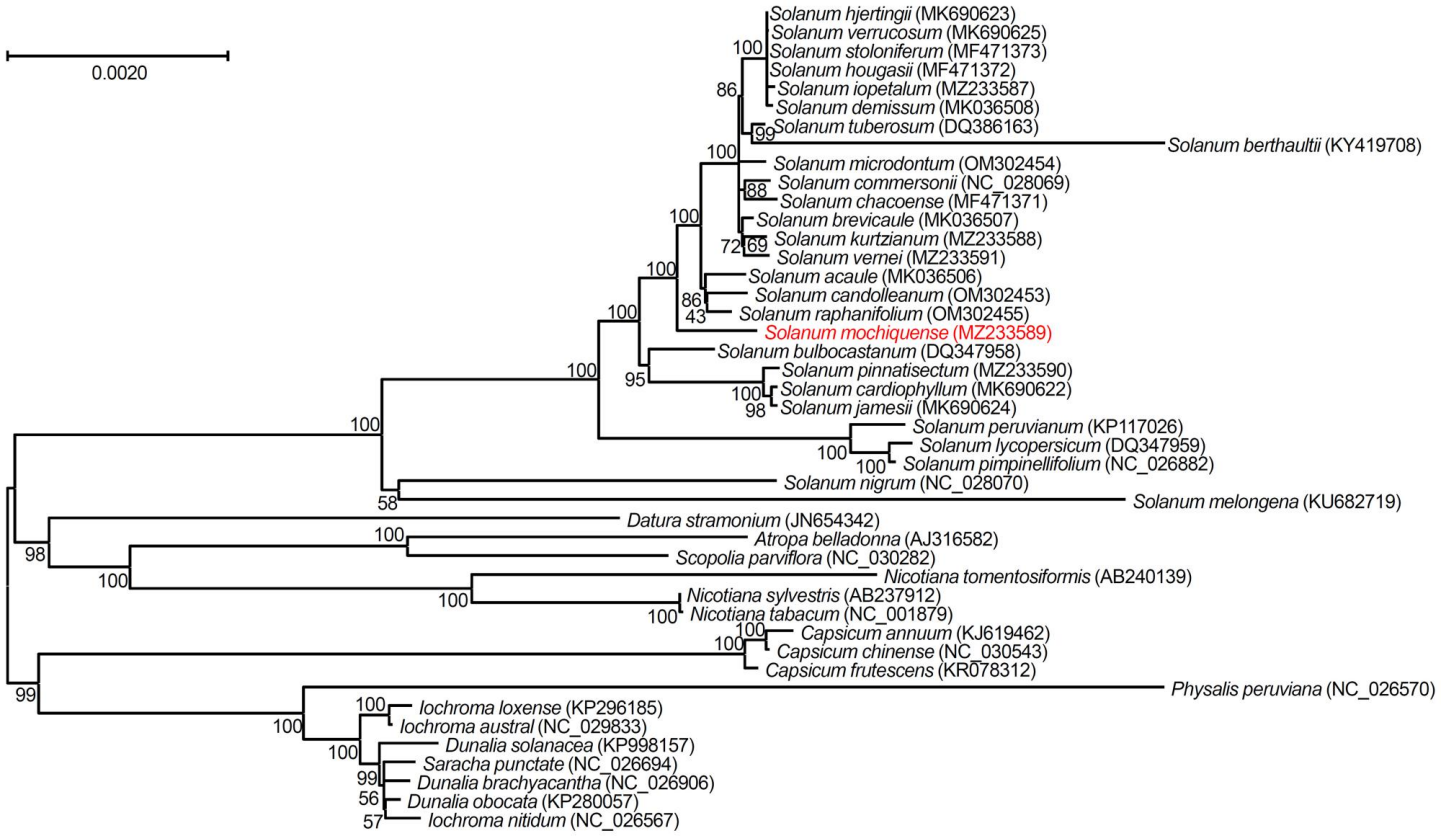
Maximum likelihood phylogenetic tree constructed by suing a general time reversible model based on the chloroplast genome sequences of *S. mochiquense* and other species belonging to the Solanaceae. Numbers in the nodes are the bootstrap values from 1,000 replicates. The data have been partially adopted from Park (2023a). The 71 sequences of annotated protein coding genes from the following 44 complete chloroplast genome sequences were used: MK690623 (Park 2022), MK690625 (Jo and Park 2024a), MF471373 (Park 2018), MF471372 (Cho et al. 2018), MZ233587 (Park 2023a), MK036508 (Cho et al. 2019), DQ386163 (Gargano et al. 2012), KY419708 (Park 2017), OM302454, NC028069 (Cho et al. 2016), MF471371 (Cho et al. 2017), MK036507 (Park 2019), MZ233588, MZ233591, MK036506 (Park 2021), OM302453, OM302455, MZ233589 (in this study), DQ347958 (Daniell et al. 2006), MZ233590 (Son and Park 2024), MK690622 (Park 2023b), MK690624 (Jo and Park 2024b), KP117026 (Wu 2016), DQ347959 (Daniell et al. 2006), NC026882 (Wu 2016), NC028070 (Park 2016), KU682719 (Ding et al. 2016), JN654342, AJ316582 (Schmitz-Linneweber et al. 2002), NC030282 (Park and Lee 2016), AB240139 (Yukawa et al. 2006), AB237912 (Yukawa et al. 2006), NC001879 (Shinozaki et al. 1986), KJ619462 (Zeng et al. 2016), NC030543 (Park et al. 2016), KR078312 (Shim et al. 2016), NC026570, KP296185, NC029833, KP998157, NC026694, NC026906, KP280057, NC026567.

To determine the phylogenetic status of *S. mochiquense*, 43 other species in the Solanaceae family were obtained from the GenBank database. The results showed that *S. mochiquense* belongs to the large clade with *S. tuberosum* and other wild potato relatives in the genus *Solanum* and is sub-grouped with other Peruvian *Solanum* species (*S. candolleanum* and *S. raphanifolium*) in the large clade. It is also closer to wild species originating from countries neighboring Peru, such as Bolivia and Argentina (Figure 3). Sedláková et al. (2017) reported partially similar results from data with three non-coding plastid DNA loci.

## Conclusion

The chloroplast genome sequence of *S. mochiquense* was completed and characterized for the first time in this study. The length and structure of the *S. mochiquense* plastome is comparable to other wild *Solanum* species. Our findings will facilitate further research investigating more detailed breeding and evolutionary aspects.

## Supplementary Material

Supplementary Figure1 mapped read depth.jpg

Supplementary Figure2 cis trans splicing genes.jpg

## Data Availability

The genome sequence data that support the findings of this study are openly available from NCBI (https://www.ncbi.nlm.nih.gov/) under accession number MZ233589. The associated BioProject, SRA, and BioSample numbers are PRJNA729868, SRR14534353, and SAMN19184899, respectively.
