# Supplementary figures and images for: Complete chloroplast genome sequence of Solanum mochiquense, one of the tuber-bearing potato relatives
Source: Mitochondrial DNA B Resour. 2024 Nov 21;9(11):1586–91. doi: 10.1080/23802359.2024.2432357 (PMC11583358; doi:10.1080/23802359.2024.2432357)

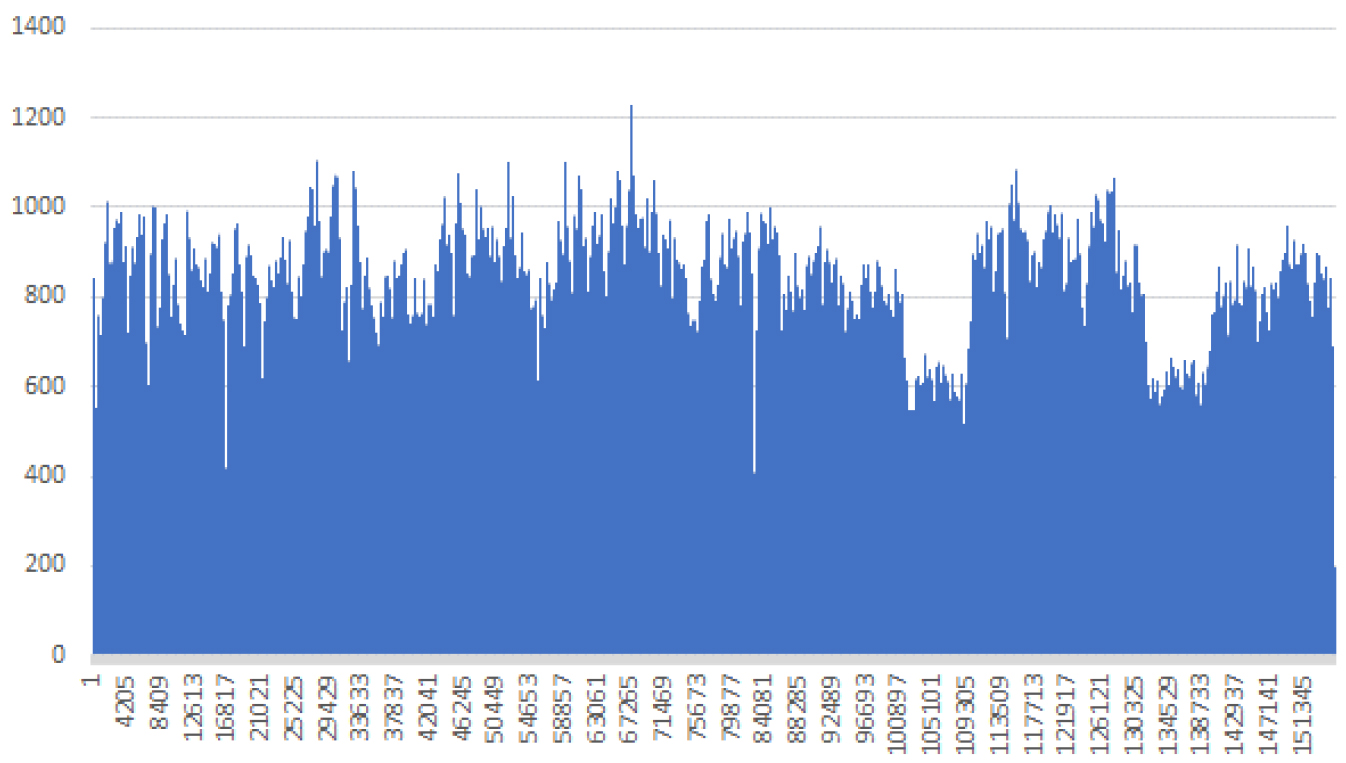

Supplement: Supplementary Figure1 mapped read depth.jpg [file TMDN_A_2432357_SM8083.jpg]

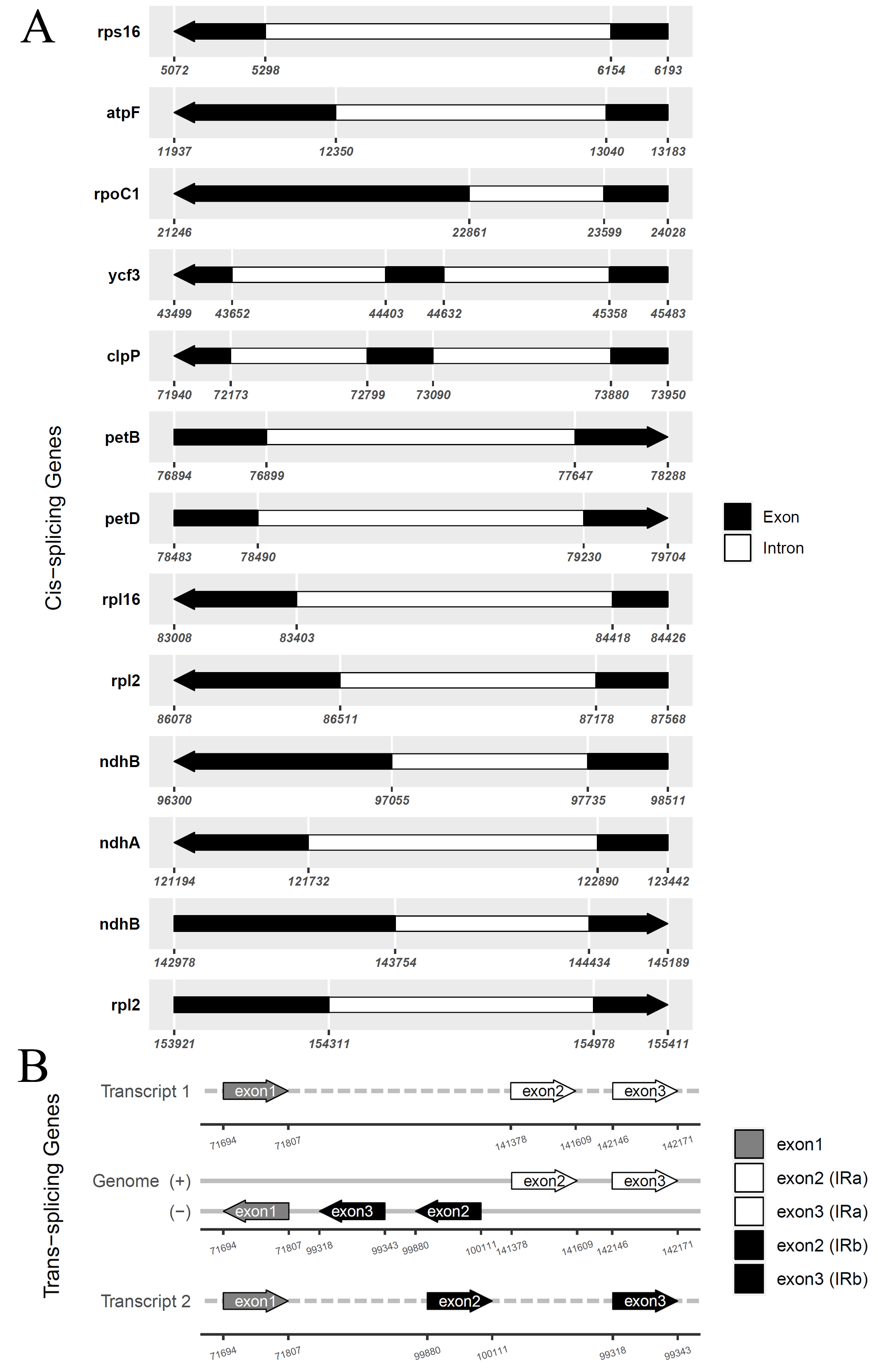

Supplement: Supplementary Figure2 cis trans splicing genes.jpg [file TMDN_A_2432357_SM8082.jpg]
